# Clinical impact of deoxynivalenol, 3-acetyl-deoxynivalenol and 15-acetyl-deoxynivalenol on the severity of an experimental *Mycoplasma hyopneumoniae* infection in pigs

**DOI:** 10.1186/s12917-018-1502-4

**Published:** 2018-06-18

**Authors:** Annelies Michiels, Ioannis Arsenakis, Anneleen Matthijs, Filip Boyen, Geert Haesaert, Kris Audenaert, Mia Eeckhout, Siska Croubels, Freddy Haesebrouck, Dominiek Maes

**Affiliations:** 1 Department of Reproduction, Obstetrics and Herd Health, Faculty of Veterinary Medicine, Ghent University, Salisburylaan 133, 9820 Merelbeke, Belgium; 2 Department of Pathology, Bacteriology and Avian Diseases, Faculty of Veterinary Medicine, Ghent University, Salisburylaan 133, 9820 Merelbeke, Belgium; 3 Department of Applied Biosciences, Faculty of Bioscience Engineering, Ghent University, Campus Schoonmeersen, Valentin Vaerwyckweg 1, 9000 Ghent, Belgium; 4 Department of Pharmacology, Toxicology and Biochemistry, Faculty of Veterinary Medicine, Ghent University, Salisburylaan 133, 9820 Merelbeke, Belgium

**Keywords:** *Mycoplasma hyopneumoniae*, Experimental, Challenge, Clinical, Impact, Deoxynivalenol, Feed, Weaned piglets

## Abstract

**Background:**

The mycotoxin deoxynivalenol (DON) is highly prevalent in cereals in moderate climates and therefore pigs are often exposed to a DON-contaminated diet. Pigs are highly susceptible to DON and intake of DON-contaminated feed may lead to an altered immune response and may influence the pathogenesis of specific bacterial diseases. Therefore, the maximum guidance level in feed is lowest in this species and has been set at 900 μg/kg feed by the European Commission. This study aimed to determine the effect of in-feed administration of a moderately high DON concentration (1514 μg/kg) on the severity of an experimental *Mycoplasma hyopneumoniae* (*M. hyopneumoniae*) infection in weaned piglets. Fifty *M. hyopneumoniae*-free piglets were assigned at 30 days of age [study day (D)0] to four different groups: 1) negative control group (NCG; *n* = 5), 2) DON-contaminated group (DON; *n* = 15), 3) DON-contaminated and *M. hyopneumoniae*-inoculated group (DONMHYO; *n* = 15), 4) *M. hyopneumoniae*-inoculated group (MHYO; n = 15). The piglets were fed the experimental diets ad libitum for five weeks and were monitored during this period and euthanized at day 35 [27 days post infection (DPI)] or 36 (28 DPI). The main parameters under investigation were macroscopic lung lesions (MLL) at euthanasia, respiratory disease score (RDS) from day 8 until day 35, histopathologic lesions and log copies of *M. hyopneumoniae* DNA detected by qPCR, determined at the day of euthanasia.

**Results:**

No significant difference was obtained for MLL at euthanasia, RDS (8–35), histopathologic lung lesions and log copies of *M. hyopneumoniae* DNA in the DONMHYO and MHYO group and consequently, no enhancement of the severity of the *M. hyopneumoniae* infection could be detected in the DONMHYO compared to the MHYO group.

**Conclusions:**

Under present conditions, the findings imply that feed contaminated with DON (1514 μg/kg) provided to weaned pigs for five weeks did not increase the severity of an experimental *M. hyopneumoniae* infection. Further research is needed to investigate the impact of DON on *M. hyopneumoniae* infections in a multi-mycotoxin and multi-pathogen environment.

## Background

The mycotoxin deoxynivalenol (DON) is a fungal metabolite produced mainly by *Fusarium graminearum* and *Fusarium culmorum* [1, 2]. *Fusarium-*produced mycotoxins, of which by toxicological viewpoint the trichothecenes DON and T-2 toxin, zearalenone and fumonisins are the most important [3], have been reported worldwide in many cereal-based cropping systems. *Fusarium s*pecies have traditionally been associated with temperate cereals, as these fungi require lower temperatures for growth and mycotoxin production [4]. Indeed, DON is one of the most common natural mycotoxin contaminants of wheat and other small cereal grains harvested in moderate climate zones [5–7]. Extensive data on global mycotoxin occurrence showed that 59% of 5819 samples of animal feed tested positive on DON presence [3, 4, 8]. In low doses, DON causes anorexia, decreased weight gain and immune stimulation [2]. Pigs are known to be a very sensitive animal species to DON, mainly because of the high oral bioavailability and differences in metabolism of this mycotoxin compared to other species [2]. In moderate to high doses (above 840 μg/kg feed), decreased feed intake or feed refusal, vomiting and immune suppression are seen [2, 9, 10]. In fact, the EU (European Union) recommended maximum pig feed guidance level for DON is 900 μg/kg, which is the lowest one compared to other farm animal species for total diets and compounds of total diets. For adult ruminants and poultry a maximum of 5000 μg/kg and 2000 μg/kg for calves and lambs are set as guidance levels [11]. On top of that, the pig consumes a cereal rich diet and DON is frequently detected in wheat, barley, corn and by-products [9, 12].

It is known that DON can have an impact on the pathogenesis of several bacterial diseases [3, 13, 14]. Exposing porcine ileal loop tissue to a DON-concentration of 1 μg/ml, potentiated the inflammatory response and significantly enhanced *Salmonella* Typhimurium invasion in and passage of the bacterium across the intestinal epithelium [15]. Furthermore, DON (0.025 μg/ml) induced an enhanced uptake of *Salmonella* Typhimurium in porcine macrophages, indicating the capacity of DON to modulate the innate immune system, and thus to increase the susceptibility of the pig to *Salmonella* Typhimurium infections [16]. Deoxynivalenol might, due to the immunomodulatory effect on the host and/or the immediate impact on the pathogen, have an impact on the course of respiratory infectious diseases in swine. However only limited in vivo information is available. A three-week ingestion period of feed contaminated with high levels of 2500 μg/kg and 3500 μg/kg DON resulted in a higher viremia and lung viral load in case of a Porcine Circovirus type 2 (PCV2) infection, and a lower body weight gain, more lung lesions and mortality in porcine reproductive and respiratory syndrome virus (PRRSv)-infected pigs, respectively [17, 18]. Pigs receiving fumonisin B1 in a concentration of 10,000 μg/kg feed, and dually infected with *Bordetella bronchiseptica* (*B. bronchiseptica*) and *Pasteurella multocida*, (*P. multocida*) were at greater risk to develop pneumonia and had an increase of the extent and severity of the pathological changes compared to dually infected pigs that did not receive fumonisin B1 [19]. Oral gavage of *P. multocida*-infected pigs with a crude extract of fumonisin B1 in a concentration of 500 μg/kg body weight (BW) per day for a period of seven days resulted in the pigs coughing more, in increased bronchoalveolar lavage fluid total cells, macrophages and lymphocytes, and resulted in an increased occurrence of lung lesions compared to the pigs only infected with *P. multocida* [20]. Dietary exposure to fumonisin B1 in a concentration of 12,000 μ/kg feed increased the risk on PRRSv–associated disease [21] and induced pulmonary edema which may aggravate *M. hyopneumoniae* infection [22].

*Mycoplasma hyopneumoniae* is causing tremendous economic losses in all intensive pig producing countries worldwide [23], despite many attempts to control the disease (enzootic pneumonia) through vaccination strategies and control measures. Consequently, there is a high prevalence of both *M. hyopneumoniae* infections and a high contamination rate of feed with mycotoxins, more specific DON, in Europe [8]. Therefore, the odds for a pig to ingest feed contaminated with DON, whilst simultaneously being infected with *M. hyopneumoniae* is high. The present study aimed to investigate the effect of in-feed administration of DON at a moderately high level of 1540 μg/kg feed, on the clinical course of an experimental *M. hyopneumoniae* infection with two genetically different *M. hyopneumoniae* strains in weaned piglets.

## Methods

### Study animals and experimental design

The study was compliant with all relevant institutional and European standards for animal care and experimentation. The experiment was approved by the Ethics Committee for Animal Experiments of the Faculty of Veterinary Medicine and Faculty of Bioscience Engineering, Ghent University (approval number EC2015/112). Fifty *M. hyopneumoniae*-free Rattlerow-Seghers piglets (RA-SE Genetics NV, Ooigem, Belgium) were included in the study. The herd of origin has been free of *M. hyopneumoniae* and PRRSv since 2012 based on repeated serological testing, absence of clinical signs and pneumonia lesions, and nested polymerase chain reaction (nPCR) testing on tracheobronchial swabs as previously described [24]. The gilts and sows in the herd were vaccinated against *Erysipelothrix rhusiopathiae* and Parvovirus before insemination. No vaccinations were administered to the piglets. The piglets were weaned on average at 26 days of age and moved four days later to the experimental facilities of the Faculty of Veterinary Medicine, Ghent University, Belgium. The piglets were individually identified by means of an ear tag. The study design, the different parameters and timing are summarized in Table 1. Upon arrival (D0) the piglets were randomly allocated to four different groups: 1) negative control group (NCG; *n* = 5): sham-inoculated D8, D9 + control diet, 2) DON-contaminated group (DON; *n* = 15): sham-inoculated D8, D9 and DON-diet (1514 μg/kg), 3) DON-contaminated and *M. hyopneumoniae*-inoculated group (DONMHYO; n = 15): experimentally inoculated with *M. hyopneumoniae* D8, D9 and DON-diet (1514 μg/kg), 4) *M. hyopneumoniae*-inoculated group (MHYO; n = 15): experimentally inoculated with *M. hyopneumoniae* D8, D9 + control diet. The number of 15 animals in the treatment groups enabled to find a difference of 5.26 ± 4.7 in the main parameter, namely macroscopic lung lesions (two-sided test) with 95% certainty and a statistical power of 80%. This difference is biologically relevant and was based on previous research in our research group [25]. The NCG (5 pigs) was used to verify whether the purchased piglets remained *M. hyopneumoniae* negative throughout the study. The different groups were housed in four different facilities equipped with absolute filtered chambers (HEPA U15) in order to avoid cross-infection of *M. hyopneumoniae* between the different groups. The pigs had free access to drinking water and were fed ad libitum.

**Table 1:** Experimental design, sample collections and timing in the different experimental groups

Study day, D	Groups			
	NCG (*n* = 5)	DON (*n* = 15)	DONMHYO (*n* = 15)	MHYO (*n* = 15)
D0^a^	Arrival			
	Weight			
	Randomisation			
D0-D35/36^b^	Commercial feed ad libitum	DON-contaminated feed ad libitum	DON-contaminated feed ad libitum	Commercial feed ad libitum
D1-D35^c^	RDS			
D8	Sham-inoculation^d^	Sham-inoculation^d^	F7.2C^e^-inoculation	F7.2C^e^-inoculation
	Weight			
	Blood			
D9	Sham-inoculation^d^	Sham-inoculation^d^	F1.12A^f^-inoculation	F1.12A^f^-inoculation
D21	BALF			
	Blood			
D35/36^b^	Weight			
	Necropsy			
	Lung sample			
	BALF			

^a^the average age of the pigs at arrival was 26 days, ^b^all pigs of the NCG and DON group, and five pigs of MHYO were necropsied at D35. Ten pigs of MHYO and all pigs of DONMHYO were necropsied at D36, ^c^RDS was not determined at D0 and D36 because the piglets arrived later than 8 a.m. (hour of performing coughing score every day) and part of the piglets were already euthanized on D36, respectively, ^d^sham-inoculation was performed with sterile Friis medium, ^e^highly virulent strain of *M. hyopneumoniae*, ^f^low virulent strain of *M. hyopneumoniae*

*NCG* negative control group, *DON* deoxynivalenol contaminated group, *DONMHYO* deoxynivalenol contaminated +*M. hyopneumoniae*-inoculated group, *MHYO* *M. hyopneumoniae*-inoculated group, *RDS* respiratory disease score, *BALF* bronchoalveolar lavage fluid

### *Mycoplasma hyopneumoniae* strains and challenge infection

The pigs were inoculated with two different strains of *M. hyopneumoniae*: a highly virulent strain F7.2C and low virulent strain F1.12. Both strains had been differentiated and characterized at proteomic level with Sodium-Dodecyl-Sulphate Polyacrylamide gelelectrophoresis (SDS-page) [26] and at genomic level with Random Amplified Polymorphic DNA (RAPD), Amplified Fragment Length Polymorphism (AFLP), PCR-Random Fragment Length Polymorphism (PCR-RFLP) of the p146 gene, Variable Number of Tandem Repeats (VNTR) analysis of p97, with Multiple-Locus of VNTR Analysis (MLVA) [27–29] and used in previous studies [24, 25, 30]. Previous research has shown that pigs are often infected with two or even three genetically different *M. hyopneumoniae* strains [29, 31, 32], and in slaughter pigs infected with different strains, more lung lesions can be detected [32]. Therefore, the pigs in the present study were inoculated with two genetically different *M. hyopneumoniae* strains, as performed previously by this research group [18], to mimic the situation in the field. All pigs were anesthetised via the intramuscular route with 0.22 ml/kg BW of a mixture of tiletamine, zolazepam (Zoletil 100®, Virbac, Louvain-la-Neuve, Belgium) and xylazine (Xyl-M® 2%, VMD, Arendonk, Belgium) and the pigs of the DONMHYO and MHYO groups were endotracheally inoculated with 7 ml of inoculum containing 10^7^ CCU/ml of strain F7.2C on D8 and 7 ml of inoculum containing 10^7^ CCU/ml of strain F1.12A on D9. On both inoculation days, the pigs of the NCG and DON group were endotracheally sham-inoculated with 7 ml of sterile Friis medium. At D35 or D36 of the experiment, the pigs were euthanized using deep anaesthesia by intramuscularly administering 0.3 ml/kg BW of a mixture of tiletamine, zolazepam (Zoletil 100®, Virbac, Louvain-la-Neuve, Belgium) and xylazine (Xyl-M® 2%, VMD, Arendonk, Belgium), followed by exsanguination. For practical reasons and to avoid *M. hyopneumoniae* contamination of the samples, all pigs of the NCG and DON group were euthanized at D35, followed by five animals of the MHYO group. All the other animals of the MHYO and all animals of the DONMMHYO group were necropsied on D36.

### Deoxynivalenol contaminated diet

A commercial antibiotic-free diet for weaned piglets was purchased (Leievoeders N.V., Waregem, Belgium). Before purchasing the feed, a sample of the batch was tested with liquid chromatography-tandem mass spectrometry (LC-MS/MS) according to Monbaliu et al. [33, 34] for the presence of DON, 3-Acetyldeoxynivalenol (3-ADON), zearalenone and fumonisin B1+ B2, the levels were below the reporting limit of 50 μg/kg, 50 μg/kg, 10 μg/kg and 50 μg/kg, respectively. The piglets of the NCG and MHYO groups were fed this commercial diet from D0 until D35/36. A part of the purchased feed (1300 kg) was transported to the laboratory of the Department of Applied Biosciences (Faculty of Bioscience Engineering, Ghent University) to add the target concentration of 1800 μg/kg feed DON. This procedure was followed: the reference strain *Fusarium graminearum (F. graminearum)* MUCL 42841 (Mycothèque de l’Université catholique de Louvain) was used to produce the DON-culture. The strain was grown in liquid mineral (MIN) medium supplemented with L-arginin as a selective nitrogen source, as previously described by Gardiner et al. [35]. After 14 days of cultivation, the culture was filtered and centrifuged. The obtained concentration of DON was determined with LC-MS/MS by adding 150 μl of the resulting undiluted MIN medium to 5 g of certified blank wheat standard (Sigma Aldrich, Overijse, Belgium). In total, 7535 mg/kg DON was quantified and 1076 mg/kg acetylated DON (3-ADON + 15-ADON) in the grown DON-culture. Next to the presence of DON, the inoculum was tested with LC-MS/MS for the presence of other *F. graminearum* trichothecenes such as nivalenol, neosolaniol, fusarenon-X, diacetoxyscirpenol, HT-2 toxin and T-2 toxin, and results were below the detection limit. Also no zearalenone was detected. Eight l of inoculum were obtained for preparation of 1300 kg of DON-contaminated feed in a concentration aimed at twice the recommended maximum pig feed level of 900 μg/kg or 1800 μg/kg DON. First, 2.67 l of the inoculum was mixed with 10 kg of feed to obtain a thoroughly mixed premix of the DON-contaminated feed. Subsequently, the premix with inoculum was added to 433.3 kg of feed in a feed mill and thoroughly mixed for at least 40 min. The same procedure was repeated twice, to obtain the total amount of 1300 kg of DON-contaminated feed in a concentration of 1800 μg/kg and the feed was collected again in the original 25 kg bags of the feeding company. After preparation of the contaminated feed, a mixed sample originating from three DON-contaminated feed bags was taken and was submitted for LC-MS/MS to obtain the true DON-concentration of the contaminated feed. The results of the LC-MS/MS of the contaminated feed were 407 ± 120 μg/kg DON, 280 ± 100 μg/kg 3-ADON and 827 ± 300 μg/kg 15-ADON, resulting in a total DON and acetylated DON (3-ADON + 15-ADON)-concentration of 1514 μg/kg in the contaminated feed sample. This feed was used in the DON and DONMHYO-groups from D0 until D35/36 of the study.

### Clinical and performance parameters

From D0 until D35/36 onwards, the piglets were observed daily at 8 a.m. for at least half an hour by the same researcher to assess appetite, faecal consistency and presence of dyspnea and tachypnea. A faecal consistency score was used to evaluate the faeces found on the pen floor before cleaning [36]: 1 (firm and shaped), 2 (soft and shaped), both addressed as a normal faecal consistency in pigs, 3 (loose) and 4 (watery), with scores 3 and 4 considered as abnormal. Daily, from D1 until D35 a respiratory disease score (RDS), ranging from 0 to 6 was recorded according to Halbur et al. (1996) [37]. Score 0 was obtained when a pig did not cough. Score 1, 3 and 5 were respectively designated as mild, moderate and severe couging after encouraged move. Score 2, 4, 6 were respectively obtained when mild, moderate and severe coughing in rest was present.The RDS was not determined at D0 and D36, as the pigs arrived later than 8 a.m. at the facilities and already part of the animals was euthanized on D35. The daily RDS values were averaged for the following periods: D1–7, D8–35/36 and D1–35/36. All pigs were weighed (kg) at the day of arrival (D0), the first inoculation day (D8) and the day of euthanasia (D35/36). The average daily gain (ADG, kg/pig/day) was calculated from D0–7, D8–35/36 and D0–35/36 by subtracting the starting weights from the final weights, divided by the number of days during that period.

### Macroscopic and histopathologic lung lesions

The lungs were removed and macroscopic lung lesions (MLL) (D35/36) were determined according to Hannan et al. (1982) [38] from each pig. Consequently, the lungs were transported to the laboratory of the Department of Pathology, Bacteriology and Avian Diseases, Faculty of Veterinary Medicine, Ghent University from the necropsy rooms from the experimental facilities in the same department and from each lung in each pig, samples from the right apical, cardiac and diaphragmatic lung lobes were collected. In case a lesion was present, a sample was collected including both healthy and affected lung tissue, at the border of the lesion. The 10% neutral formalin fixed and paraffin embedded samples were stained with hematoxylin and eosin. Using light microscopy the samples were investigated and scored for the degree of peribronchiolar and perivascular lymphohistiocytic infiltration and nodule formation (cuffing) [39]. The scoring system ranged from 1 to 5, with score 1 and 2 considered not to be related with *M. hyopneumoniae* infection as previously described [24, 39, 40].The percentage of air (percentage of lung area occupied by air) was examined by means of an automated image analysis system (Leica application suite AF Lite (Diegem, Belgium) and image J (Bethesda, Maryland, USA) [41]. This parameter is inversely proportional to the lymphohistiocytic infiltration in the lung tissue and the intrabronchiolar-and bronchial exudate [25].

### Quantitative PCR for *M. hyopneumoniae*

Two weeks post inoculation (PI) (D21) of the high virulent strain F7.2C (D8), bronchoalveolar lavage (BAL) fluid from all pigs, while conscious was collected. After snaring the pigs and opening their mouth with a gag, a catheter (Portex® Dog Catheter with Female Luer Mount, Smiths Medical International Ltd. Kent, United Kingdom) was inserted allowing to flush the lungs with 10 ml of sterile phosphate buffered saline (PBS). The PBS fluid was subsequently aspirated. At the day of necropsy (D35/36) the head bronchus of the left part of the lung was flushed with 10 ml of sterile PBS before collection of the histopathological samples. After collection, the BAL fluids were stored at − 70 °C until they were analysed. The DNA was extracted with the DNeasy Blood & Tissue kit (QIAGEN, Qiagen Benelux, B.V., Antwerp, Belgium) with the DNA Purification protocol for bloods or bloody fluids (spin protocol) on 200 μl of BAL fluid according to the manual instructions and quantitative PCR (qPCR) was performed as previously described to detect the number of *M. hyopneumoniae* organisms [42]. Briefly, after DNA-extraction, qPCR was performed with primers p102f (5’GTCAAAGTCAAAGTCAGCAAAC 3′) and p102r (5’AGCTGTTCAAATGCTTGTCC 3′) using SensiMixTM SYBR (Bioline GmbH, Luckenwalde, Germany) in the CFX384 real-time PCR detection system (Bio-Rad, Nazareth, Belgium). A tenfold dilution series of *M. hyopneumoniae* DNA of strain F7.2C was used to convert the threshold values to the number of *M. hyopneumoniae* organisms. Values below the dilution of 1.50 × 10^1^ (1.18 log copies) were considered as negative [31, 42].

### Serology

A blocking ELISA (IDEIA™ *Mycoplasma hyopneumoniae* EIA kit, Oxoid Limited, Hampshire, UK) was performed according to the instructions in the protocol manual and as previously described [24] to detect antibodies against *M. hyopneumoniae* in the blood collected at D28, D21 and at necropsy (D35/36). Sera with optical density < 50% of the average value of the OD-buffer control were considered to be positive. All values above or equal to 50% of the average value of the OD-buffer control were classified as negative .

### Routine bacteriological culture on bronchoalveolar lavage fluid

Ten μl of BAL fluid collected at necropsy (D35/36) of each pig was inoculated on Columbia agar supplemented with 5% sheep blood (Oxoid, Hampshire, UK) with a *Staphylococcus pseudintermedius* streak for bacteriological examination [43]*.* Plates were incubated for 48 h in a 5% CO_2_-enriched environment at 35 °C for identification of respiratory bacteria in the lungs. All macroscopically different colonies were identified to the species level (score value > 2.000) with a Bruker Daltonic Microflex LT Biotyper Biotyper MALDI-TOF mass spectrometer by using the direct transfer method and α-cyano-4-hydroxycinnamic acid (HCCA) as matrix, according to the manufacturer’s guidelines. The spectra were obtained and analysed with the MBT Compass software version 3.1. (Bruker Daltonik), which included a database of 6903 mean spectra projections.

### Statistical analysis

The independent variable in the statistical analyses was ‘group’: NCG, DON, DONMHYO and MHYO-group. The dependent variables were RDS, weight, ADG, MLL, histopathology and percentage of air, qPCR-results, percentage of *M. hyopneumoniae* qPCR-positive samples, *M. hyopneumoniae* specific AB expressed in OD values and percentage of ELISA *M. hyopneumoniae* positive samples. These variables were all run in separate models with ‘pig’ as statistical unit and no additional factors included into the model. The normality of the data was investigated by means of descriptive statistics, except for the binary data (*M. hyopneumoniae* qPCR positive samples and ELISA *M. hyopneumoniae* positive samples). The parameters BW, ADG and percentage air analysis were normally distributed and a one-way analysis of variance (ANOVA) test was used. In case of the RDS, a repeated measures ANOVA was performed. Scheffé’s post-hoc test was used to make pair-wise comparisons. The qPCR-results and *M. hyopneumoniae* specific antibodies were not normally distributed and therefore, a non-parametric Kruskal-Wallis test was used, with the Dunn-Bonferroni approach to make pair-wised comparisons, as well for MLL and histopathology results. In case of the normally distributed data, the mean and standard deviation (SD) were reported, in case of the non-parametric data, the median and the interquartile range were reported. All analyses for these parameters were performed with SPSS 23 for Windows (SPSS inc. Illinois, USA). Percentage of seropositive pigs and percentage of pigs testing positive with qPCR in each group were analyzed using binomial logistic regression (R version 3.3.1) [44]. The results were considered to be statistically significant when *P* < 0.05.

## Results

### Clinical and performance parameters

**Fig. 1:**
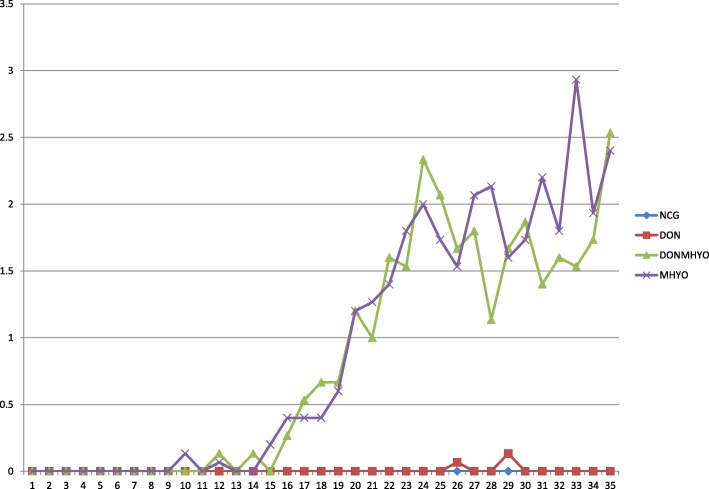
Course of average respiratory disease score (RDS) from day 1 until the day of necropsy (D 35). Average RDS from D1 until D35 in the negative control group (NCG), the DON-contaminated group (DON), DON-contaminated and *M. hyopneumoniae*-inoculated group (DONMHYO), and *M. hyopneumoniae*-inoculated group (MHYO). The challenge infections were performed on D8 (a highly virulent *M. hyopneumoniae* strain F7.2C) and D9 (a low virulent strain F1.12) in DONMHYO and MHYO. The NCG and DON were sham-inoculated with sterile Friis medium on D8 and D9

In none of the groups, feed refusals or vomiting were observed. No tachypnea, nor dyspnea were observed in any of the groups. Post-weaning diarrhoea was observed from D0 onwards until D3 in all groups, therefore all pigs were treated IM with Colistin sulphate (Colivet ‘S’, Prodivet, Eynatten, Belgium) from D0 onwards for 5 days, according to the product leaflet. The post weaning diarrhoea lasted until D8, D3, D3 and D7 in NCG, DON, DONMHYO and MHYO, respectively. In three groups some pigs with faecal consistency score 3 were noticed throughout the study: in NCG two pigs at D20 and D23 respectively, in DON one pig at D13 and in DONMHYO two pigs at D20 and D23, respectively. In MHYO, normal faecal consistency was observed throughout the study. Coughing was not observed in the NCG. All results of the different time periods for this parameter in the study are shown in Table 2 (RDS _1–7_, RDS _8–35_ and RDS _1–35_). The RDS from the first inoculation day onwards until euthanasia (RDS _8–35_) were 0 ± 0, 0.0071 ± 0.028, 1.04 ± 0.82, 1.14 ± 0.92 for NCG, DON, DONMHYO and MHYO, respectively (*P* < 0.001). No statistically significant differences were obtained between all groups for D1–7. For D8–35 and D1–35 a statistically significant difference was obtained between the experimentally infected (DONMHYO and MHYO) and non-infected pigs (NCG and DON) (*P* < 0.001). However, no statistically significant differences were obtained for DONMHYO and MHYO groups in each time period of the study (Table 2). The daily course of RDS _1–35_ for each group is shown in Fig. 1.

**Table 2:** Results of the clinical parameters, macroscopic and microscopic lung lesions in the different experimental groups

Parameter	Groups				
	NCG (*n* = 5)	DON (*n* = 15)	DONMHYO (*n* = 15)	MHYO (*n* = 15)	*P*-value
RDS					
D1–7	0 ± 0	0 ± 0	0 ± 0	0 ± 0	1.00
D8–35	0 ± 0^a^	0.01 ± 0.03^a^	1.04 ± 0.82^b^	1.14 ± 0.92^b^	< 0.001
D1–35	0 ± 0^a^	0.01 ± 0.03^a^	0.83 ± 0.84^b^	0.91 ± 0.94^b^	< 0.001
Weight ± SD (kg)					
D0	6.37 ± 1.09	6.39 ± 0.92	6.38 ± 1.04	6.42 ± 1.23	1.00
D8	7.70 ± 1.13	8.04 ± 1.27	8.20 ± 1.08	8.11 ± 1.90	0.92
D35/36	20.10 ± 2.33	19.45 ± 3.83	21.02 ± 3.18	21.75 ± 4.36	0.38
ADG (kg/pig/day)					
D0–8	0.52 ± 0.14	0.53 ± 0.19	0.56 ± 0.15	0.67 ± 0.17	0.12
D0–35/36	0.39 ± 0.06	0.37 ± 0.11	0.41 ± 0.08	0.43 ± 0.10	0.41
D8–35/36	0.46 ± 0.08	0.42 ± 0.13	0.46 ± 0.10	0.49 ± 0.10	0.37
MLL, histopathology and percentage of air D35/36					
MLL	0 ± 0^a^	0 ± 0^a^	2.77 ± 3.22^b^	5.87 ± 7.32^b^	< 0.001
Histopathology	1.70 ± 0.20^a^	2.00 ± 0.30^a^	2.40 ± 0.80^b^	2.40 ± 0.90^b^	< 0.001
Percentage of air (%)	45.94 ± 6.54	46.85 ± 6.86	49.75 ± 9.90	46.96 ± 8.51	0.25

Respiratory disease score (RDS), bodyweight, average daily weight gain (ADG), macroscopic lung lesions (MLL), histopathology score of the lungs and percentage of air in the lungs for NCG, DON group, DONMHYO group, MHYO group

The parameters bodyweight, ADG, percentage of air analysis were analysed by means of one way analysis of variance, the parameter RDS with a repeated measures analysis of variance, in both cases with Scheffé post-hoc test to make pair-wised comparisons, therefore means ± SD are reported. The parameter MLL and histopathology were analysed with Kruskal-Wallis test and the Dunn-Bonferroni approach to make pair-wised comparison, therefore medians and interquartile range are reported. Different superscripts in one row are statistically different (*P* < 0.05)

*NCG*: negative control group, *DON*: deoxynivalenol contaminated group, *DONMHYO*: deoxynivalenol contaminated +*M. hyopneumoniae*-inoculated group, *MHYO*: *M. hyopneumoniae*-inoculated group, *SD*: standard deviation, *n* number, *D* Day of the study, *ADG* average daily gain, *RDS* respiratory disease score, *MLL* macroscopic lung lesions

There were no significant differences between the groups for the parameter BW and the ADG during the different time periods (Table 2).

### Macroscopic and histopathologic lung lesions

The MLL of the NCG, DON, DONMHYO and MHYO groups were 0 ± 0, 0 ± 0, 2.77 ± 3.22 and 5.87 ± 7.32, respectively (*P* < 0.001). The histopathological lung lesions were 1.70 ± 0.20, 2.00 ± 0.30, 2.40 ± 0.80 and 2.40 ± 0.90 for the NCG, DON, DONMHYO and MHYO group, respectively (*P* < 0.001). The percentage of air was 45.94 ± 6.54, 46.85 ± 6.86, 49.75 ± 9.90 and 46.96 ± 8.51 for NCG, DON, DONMHYO and MHYO, respectively (*P* = 0.25). There were no statistically significant differences between the DONMHYO and MHYO groups for these above-mentioned parameters, however there were statistically significant differences between the experimentally infected (DONMHYO and MHYO) and non-infected pigs (NCG and DON) (*P* < 0.001), except for the parameter percentage of air analysis (*P* = 0.25) (Table 2).

### Quantitative PCR for *M. hyopneumoniae*

The samples of the NCG and DON group remained negative throughout the study. The qPCR results at D21 were 0.05 ± 0.37, 0.63 ± 0.81, 3.92 ± 2.60 and 3.24 ± 1.91 (*P* < 0.001) and at D35/36 0.80 ± 0.70, 0.29 ± 1.09, 4.05 ± 1.38 and 4.20 ± 1.00 (*P* < 0.001) for NCG, DON, DONMHYO and MHYO, respectively. There were no significant differences between the DONMHYO and MHYO group, however there were statistically significant differences between the experimentally infected (DONMHYO and MHYO) and non-infected pigs (NCG and DON) (*P* < 0.001) for D21 and D35/36 (Table 3).

**Table 3:** Results of *M. hyopneumoniae*-DNA detection in the BALF and serology in the different experimental groups

Parameter	Groups				
	NCG (*n* = 5)	DON (*n* = 15)	DONMHYO (*n* = 15)	MHYO (*n* = 15)	*P*-value
qPCR (log copies of *M. hyopneumoniae* DNA /ml BALF) ± SD					
D21	0.05 ± 0.37^a^	0.63 ± 0.81^a^	3.92 ± 2.60^b^	3.24 ± 1.91^b^	< 0.001
D35/36	0.80 ± 0.70^a^	0.29 ± 1.09^a^	4.05 ± 1.38^b^	4.20 ± 1.00^b^	< 0.001
Percentage of *M. hyopneumoniae* qPCR positive samples (n of positive samples/total n of samples tested)					
D21 (%)	0 (0/5)^a^	0 (0/15)^a^	87 (13/15)^b^	93 (14/15)^b^	< 0.001
D35/36 (%)	0 (0/5)^a^	0 (0/15)^a^	100 (15/15)^b^	100 (15/15)^b^	< 0.001
*M. hyopneumoniae* specific AB expressed in OD-values ± SD					
D8	1.68 ± 0.23	1.83 ± 0.34	1.71 ± 0.19	1.70 ± 0.19	0.27
D21	1.15 ± 0.22^a^	1.29 ± 0.23^a^	1.16 ± 0.36^ab^	0.94 ± 0.26^b^	0.001
D35/36	1.15 ± 0.27^a^	1.16 ± 0.18^a^	0.25 ± 0.18^b^	0.23 ± 0.10^b^	< 0.001
Percentage of ELISA *M. hyopneumoniae* positive samples (n of positive samples/total n of samples tested)					
D8 (%)	0 (0/5)	0 (0/15)	0 (0/15)	0 (0/15)	1.00
D21 (%)	0 (0/5)	0 (0/15)	0 (0/15)	13 (2/15)	0.17
D35/36 (%)	0 (0/5)^a^	0 (0/15)^a^	100 (15/15)^b^	100 (15/15)^b^	< 0.001

Respiratory disease score (RDS), bodyweight, average daily weight gain (ADG), macroscopic lung lesions (MLL), histopathology score of the lungs and percentage of air in the lungs for NCG, DON group, DONMHYO group, MHYO group

The parameter qPCR and *M. hyopneumoniae* specific AB were analysed with a non-parametric Kruskal-Wallis and the Dunn-Bonferroni approach to make pair-wised comparisons, therefore median and interquartile range are reported. The prevalence of *M. hyopneumoniae* qPCR positive samples and the prevalence of ELISA *M. hyopneumoniae* positive samples were analysed with binomial logistic regression. Different superscripts in one row are statistically different (*P* < 0.05)

*NCG*: negative control group, *DON*: deoxynivalenol contaminated group, *DONMHYO*: deoxynivalenol contaminated + *M. hyopneumoniae*-inoculated group, *MHYO*: *M. hyopneumoniae*-inoculated group, Different superscripts in one row are statistically different (*P* < 0.05), *n* number, *SD* standard deviation, *D* Day of the study, *M. hyopneumoniae*: *Mycoplasma hyopneumoniae,* *AB* antibodies, *OD* optical densities

### Serology

The serological results are presented in Table 3. All pigs of the NCG and DON remained serologically negative throughout the study. The OD-values of the serological results at D35/36 were 1.15 ± 0.27, 1.16 ± 0.18, 0.25 ± 0.18 and 0.23 ± 0.09 for the NCG, DON, DONMHYO and MHYO, respectively. At necropsy (D35/36), all pigs of DONMHYO and MHYO were serologically positive.

### Routine bacteriological culture on bronchoalveolar lavage fluid

Few colonies of *Streptococcus suis* were isolated from one pig of both the DONMHYO (1/15) and MHYO (1/15) groups. In addition, few colonies of *B. bronchiseptica* were isolated from one pig of DON (1/15) and four pigs of MHYO (4/15).

## Discussion

In the present study, the outcome of the main parameters (RDS, MLL, histopathological lesions and log copies of *M. hyopneumoniae* DNA in bronchoalveolar lavage fluid) was not statistically different in *M. hyopneumoniae* infected pigs that received or did not receive DON-contaminated feed. This indicates that ingestion of DON-contaminated feed at a concentration of 1514 μg/kg, exceeding the maximum guidance level for pigs according to EU regulation, did not aggravate the severity of an experimental *M. hyopneumoniae*-infection in weaned piglets.

The challenge infection was successful as all animals in the *M. hyopneumoniae* inoculated groups (MHYO and DONMHYO group) coughed, showed lung lesions (except for one animal in the MHYO group), seroconverted and *M. hyopneumoniae*-DNA was detected in BAL fluid at necropsy. The results obtained in the *M. hyopneumoniae* challenged pigs (DONMHYO and MHYO group) differed significantly from the non-challenged pigs (DON group, NCG). The latter pigs remained serologically negative throughout the entire study period and no *M. hyopneumoniae*- DNA was detected in BAL fluid two weeks post-inoculation, nor at necropsy. The obtained data in the challenged groups were comparable with a previous study performed by our research group with the same challenge model [24], demonstrating the repeatability of the model. The two *M. hyopneumoniae*-strains were not administered on the same day, as the infection model practiced at our research group uses seven ml inoculum containing 10^7^ CCU of the strain/ml [31]. It is not known whether piglets of four to five weeks of age (on average 26 days, D0 at arrival and inoculation at D8) are able to cope with twice the inoculum volume (14 ml for both strains). In addition, it is not known whether both strains will grow next to each other, or whether one strain will overgrow the other strain consuming most of the nutrients, when these *M. hyopneumoniae*-strains are grown in the same culture flask. Consequently, it was decided to challenge the piglets on two consecutive days.

In the present study, DON was aimed to be administered at two times the maximum guidance level of DON advised by the European commission for pigs (1800 μg/kg feed) [11]. This dose was selected as Madson et al. [45] stated that moderate (1000–5000 μg/kg feed) to high concentrations (> 5000 μg/kg feed) are associated with delayed or supressed immune responses due to leukocyte apoptosis and resulting in increased disease susceptibility [45, 46]. It was demonstrated that a dose-related decrease in daily feed intake is observed when administering DON-supplemented feed to pigs, and that this effect was mostly significant to the control feed in the range of 2000 μg/kg – 4000 μg/kg [47–49]. Therefore, a dose below this range was chosen to be administered to the pigs in this study to limit feed refusals and to avoid hampering of DON possibly influencing the investigated parameters. The commercial, un-spiked feed was tested before the start of the study, not only for the presence of DON, but also for zearalenone and fumonisin B1 + B2, to avoid these components having an effect in the feed of the control groups (MHYO group, NCG) and avoiding an additional or synergistic effect to the DON-toxicity in the DON-spiked groups (DON group, DONMHYO group) [46]. After preparation of the contaminated feed, a mixed feed sample from three DON-contaminated feed bags was submitted to LC-MS/MS to detect the DON-concentration and to ensure thorough mixing of the DON-inoculum in the feed. The results of the LC-MS/MS was 1514 μg/kg of DON and acetylated forms and deviated only slightly from the target concentration of 1800 μg/kg. Cytotoxicity of the produced DON in the study was not tested on beforehand, as the deleterious effects of DON in the pigs are known and the same DON inoculation method in pig feed was already successfully used in a study by Goossens et al. [50]. In the latter study, the in vivo effect of DON on intestinal damage influencing the resorption of doxycycline was determined in pigs.

It remains to be elucidated why no impact of DON in the pigs was observed in the present study. The degree of susceptibility of certain breeds/lines to the effects of DON might be one explanation [51]. For instance a lower severity in porcine circovirus type 2 associated histopathological lesions has been shown for Piétrain compared to landrace pigs [52], but so far no studies have assessed the impact of genetic differences in sensitivity to the effects of DON.

No feed-refusals were observed in the pigs administered the DON-contaminated feed. The minimum emetic dose for orally distributed DON in pigs is 100 μg/kg [53], yet vomiting was not observed in this study. The reason why this did not occur is unclear. It must be noted that the observation period of the pigs (30 min. of RDS-scoring, 15 min. Feeding and cleaning in the morning, 10 min. of observation and cleaning in the evening) was fairly short compared to the time the pigs spent in the facilities. However, the main researcher was always present after providing the feed to identify possible sick pigs, as most healthy pigs start eating immediately after providing the feed and Young et al., [54] observed that vomiting occurs within minutes after ingesting the DON-contaminated feed. Pestka et al. [53] stated that vomiting due to DON-contaminated feed, is more likely if the feed is ingested at once and not via smaller portions throughout the day. The contaminated feed and hence the DON in the DONMHYO and DON pigs, was consumed throughout the day as the pigs were fed ad libitum and had freely access to the feed.

The *M. hyopneumoniae*-infected pigs administered the DON-supplemented feed did not have a higher RDS compared to the pigs only infected with *M. hyopneumoniae*. No other studies are available investigating the impact of DON on respiratory tract disease signs in *M. hyopneumoniae*-infected pigs. However the effect of fumonisin B1 in combination with *M. hyopneumoniae* or the effect of DON on other pathogens has been studied. Pósa et al., [22] studied the effect of 20,000 μg/kg fumonisin B1 on *M. hyopneumoniae*-infected pigs, however no firm conclusions could be drawn regarding the difference in coughing between the *M. hyopneumoniae*-infected pigs with or without fumonisin B1 supplementation in the feed. Halloy et al. [20] investigated the impact of fumonisin B1 administered orally (500 μg/kg BW per day, seven days) in *P. multocida*-infected pigs and concluded that these pigs coughed more compared to the pigs only infected with *P. multocida*.

It is known that *M. hyopneumoniae* infections can negatively influence production parameters, such as ADG [55]. Deoxynivalenol ingestion in pigs may result in reduced feed intake, and subsequently decrease ADG [51, 56, 57]. In the present study, DON did not decrease ADG in the *M. hyopneumoniae* inoculated animals. This result is in agreement with Accensi et al. [9] (840 μg DON/kg feed), Gerez et al. [46] (1500 μg DON/kg feed), and Savard et al. [17]. The latter authors studied the impact of DON (2500 and 3000 μg DON/kg feed) in pigs that were simultaneously infected with PCV2. Pósa et al., [22], did not find an effect of fumonisin B1 (20, 000 μg/kg) on ADG in *M. hyopneumoniae*-infected pigs. Rotter et al. [58] observed an adaptation of pigs to oral ingestion of *Fusarium* mycotoxins from one week onwards. In that study, pigs were fed a diet mixed with naturally contaminated corn (28,700 μg/kg DON, 8600 μg/kg 15-ADON and1,100 μg/kg ZEA) to obtain DON-concentrations of 750, 1500 or 3000 μg DON /kg feed during 28 days. During the first week, the exposed pigs had lower weight gains than the pigs fed a non-contaminated control feed. At the end of the study, however, the overall weight gains did not differ anymore between these groups. This adaptation might be one of the reasons why we did no see an effect of DON on daily growth. The relative low number of pigs, followed up during a limited period of time, which is inherent in experimental infection studies like ours, also makes it difficult to obtain a statistical significant difference in daily growth [31, 59, 60]. No effect of DON ingestion on the macroscopic and histopathological lung lesions was observed in the *M. hyopneumoniae* infected group. This finding is in agreement with Pósa et al. [19], who neither saw a statistical difference in macroscopic lung lesions between the *B. bronchiseptica* and *P. multocida* dually-infected groups with or without supplementation of fumonisin B1 in the feed (10,000 μg/kg) of three-day-old piglets. Savard et al. (2015) on the other hand, did observe an effect of DON-supplementation in the feed (3500 μg/kg, three weeks) on macroscopic lung lesions in PRRSv-infected pigs [51]. In this study, DON-ingestion did not influence the number of log copies detected in the *M. hyopneumoniae* infected animals. Similarly, Savard et al. [61] did not observe an effect of DON-contamination on the presence of viral RNA, measured with qPCR, in PRRSv-infected pigs. It is not clear why the obtained effects of dietary DON vary among experiments, It might be explained by different factors such as starting weight or age of the pigs, the contamination source of DON (natural versus artificial contamination), presence of other known or unknown undetected fungal metabolites or pathogens, duration of the study (adaptation), number of pigs used in the study, gender of the pigs, health status, nutritional balance of the pig and statistical design of the study [10, 62, 63]. It is not known why DON did not influence a *M. hyopneumoniae* infection under the circumstances in this study. Deoxynivalenol has a good distribution in the lung of the pig [64]. However, as *M. hyopneumoniae* is attached to the cilia of the upper respiratory tract and does not invade the parenchyma of the lung [23, 65, 66], it might be that DON is not able to exert its effect on the pathogen. More research is needed to investigate this relationship in pigs, for instance by in vitro tests on pig tracheal explants. On the other hand discrepancies have been reported between in vivo (higher viremia in pigs exposed to DON in the feed) and in vitro (decreased PRRSv replication in MARC-cells) effects of DON on PRRSv [18, 61], thus extrapolation of in vitro results to in vivo effects has to be done cautiously.

## Conclusions

No effect was observed of DON contamination in a moderately high dose in the feed on the severity of an experimental *M. hyopneumoniae* infection in weaned piglets. In the field, however, the impact of DON-contaminated feed on a *M. hyopneumoniae*-infection might be more expressed, because mostly multi-mycotoxin contamination of the feed occurs [3, 52–54], the pigs can be exposed to DON-contaminated feed for a longer period than the five week exposure period in this study, often suboptimal housing and climate conditions may prevail and other pathogens may be present [55, 56]. Further research should assess the impact of DON on *M. hyopneumoniae* infections under these multi-pathogen and multi-mycotoxins circumstances and investigating the impact of DON in vitro on *M. hyopneumoniae* in tracheal explant cells. More research could also focus on factors influencing the effect on DON such as health status, gender, age, and possible genetic resistance of the pigs.

## Abbreviations

3-ADON: 3-Acetyldeoxynivalenol
ADG: average daily gain
ANOVA: one way analysis of variance
BAL: bronchoalveolar lavage
BW: body weight
D: study day
DON: deoxynivalenol
DON: DON-contaminated
DONMHYO: DON-contaminated and *M. hyopneumoniae*- inoculated
EU: European Union
*F. graminearum*: *Fusarium graminearum*
LC-MS/MS: liquid chromatography-tandem mass spectrometry
MHYO: *M. hyopneumoniae*- inoculated
MIN: liquid mineral
MLL: macroscopic lung lesions
NCG: negative control group
nPCR: nested polymerase chain reaction
PBS: phosphate buffered saline
PI: post inoculation
qPCR: quantitative PCR
RDS: respiratory disease score
